# Myeloid PDLIM2 repression as a common mechanism of infection susceptibility in lung diseases

**DOI:** 10.3389/fimmu.2025.1669117

**Published:** 2025-11-19

**Authors:** Feng Gao, Xujie Liu, Fan Sun, Yadong Xiao, Gutian Xiao, Zhaoxia Qu

**Affiliations:** 1 Department of Molecular Microbiology and Immunology, Hastings Center for Pulmonary Research, Norris Comprehensive Cancer Center, University of Southern California Keck School of Medicine, Los Angeles, CA, United States; 2 Department of Microbiology and Molecular Genetics, UPMC Hillman Cancer Center, University of Pittsburgh School of Medicine, Pittsburgh, PA, United States

**Keywords:** PDLIM2, NF-κB, RelA/p65, lung infection, COPD, ILD/IPF, lung cancer, LPS

## Abstract

**Introduction:**

The PDZ-LIM domain-containing protein PDLIM2 serves as a unique tumor suppressor and immune modulator. Its repression in either lung epithelial or myeloid cells has been shown to promote lung cancer and therapy resistance. However, whether PDLIM2 plays a broader role in other lung diseases remains unclear.

**Methods:**

Gene expression data on human samples were exploited to investigate if PDLIM2 is repressed in the lung of patients with chronic obstructive pulmonary disease (COPD) or interstitial lung disease (ILD/idiopathic pulmonary fibrosis (IPF). PDLIM2 conditional knockout (KO) mice and wild type (WT) control mice were intratracheally instilled with the bacterial endotoxin lipopolysaccharide (LPS) to induce acute lung injury (ALI), a murine model of human acute respiratory distress syndrome (ARDS) that can also provide mechanistic insights into COPD, pulmonary fibrosis (PF) and infectious disease. Kaplan-Meier estimator was used to determine animal survival rate, and histological analysis and single-cell RNA sequencing (scRNA-seq) of mouse lung tissues were performed to systematically define the roles of PDLIM2 at the population and single-cell level. *Ex vivo* phagocytosis and neutrophil extracellular trap (NET) formation assays were also performed to validate the scRNA-seq analysis.

**Results:**

PDLIM2 was repressed in the lungs of COPD and ILD/IPF patients, and this repression was associated with disease severity. Selective deletion of PDLIM2 in myeloid cells rendered mice more vulnerable to lung injury and mortality by LPS intratracheal instillation. The increased susceptibility was linked to exacerbated pro-inflammation signaling and diminished anti-inflammation signaling in the lung, and particularly, in lung macrophages and neutrophils.

**Conclusions:**

PDLIM2 plays an indispensable role in preventing ALI/ARDS and death, and its repression is associated with COPD and ILD progression. These data suggest that PDLIM2 repression, especially in lung myeloid cells, is a common mechanism driving COPD, ILD/IPF, and lung cancer and increasing patients’ susceptibility to infection.

## Introduction

COPD and lung cancer are the leading causes of respiratory mortality and cancer death, respectively (1, 2). On the other hand, ILD is a term for a large group of lung diseases characterized by fibrosis and scarring in the lung, with IPF as the most common and severe form (3–5). While those fatal diseases are fundamentally different, they are often associated with aberrant lung inflammation (6–9). Moreover, they all make patients much more susceptible to lung infection (10–13). Interestingly, both COPD and ILD/IPF are significant independent risk factors and have long been assumed to be pre-stage diseases and causative drivers for lung cancer (6, 14–18). However, the common mechanisms and molecular links for these malignancies remain largely unknown. As a matter of fact, our knowledge of either COPD or ILD/IPF is poor, and accordingly, the treatment options and outcomes for those incurable diseases are very limited.

In this regard, the immune modulator and tumor suppressor PDLIM2, which is expressed highest in the lung under normal conditions, is repressed in more than 90% of all human lung cancer cases when 50% of the expression level of lung tissues adjacent to tumors is used as the cut-off (19–30). Human and mouse studies demonstrate PDLIM2 repression as a causative driver of lung cancer and therapy resistance (20, 21). For example, lung epithelial- or myeloid-specific PDLIM2 deletion or global PDLIM2 deletion in mice promotes lung cancer development, chemoresistance, and/or causes complete resistance to immune checkpoint inhibitors (ICIs) (20–22). Remarkably, clinically feasible nano-delivery of PDLIM2 (nanoPDLIM2) shows a promising efficacy as a monotherapy, and in combination with ICIs and chemo drugs, completely eradicates all tumors in most animals without adding toxicity, in the preclinical model of refractory lung cancer (21, 31). It is thus both scientifically and clinically important to investigate the roles of PDLIM2 in COPD, ILD/IPF, and lung infectious diseases.

## Materials and methods

### Animals and LPS intratracheal instillation

PDLIM2^flox/flox^/Lysozyme M-Cre^+/-^ (PDLIM2 mKO), PDLIM2^flox/flox^, Lysozyme M-Cre^+/-^, and WT mice have been described before (20–22, 32–34). All mice were under a pure FVB/NJ background. Mice of 6-8-week-old were intratracheally instilled with LPS (4 mg/g body weight, Sigma-Aldrich) or phosphate-buffered saline (PBS), followed by daily body weight monitoring. When the weight loss of an infected mouse was more than 20% of the initial body weight, the mouse was humanely euthanized and counted as dead. Some mice were euthanized for bronchioalveolar lavage (BAL) collection, lung histological analysis, and scRNA-seq assay at 48 hours after LPS treatment. All animals were maintained under a specific pathogen-free condition and used according to protocols approved by the IACUC of the University of Pittsburgh and the University of Southern California.

### Lung histological assays

Lung tissues were excised, fixed in formalin, embedded in paraffin, and cut into 4-5μm thick sections. Sections were stained with H&E or Trichrome, and images were analyzed using ImageJ software (NIH). The histological lung injury was scored according to the standard scoring system (35, 36), which includes four parameters: (A) alveolar space neutrophils; (B) interstitial neutrophils; (C) proteinaceous debris in alveolar space; and (D) alveolar septal thickening. The total score was calculated as: ((20 x A) + (14 x B) + (7 x C) + (2 x D))/86. A total of 20 random high-power fields (400x total magnification) of each mouse were scored by the blinded researcher. The final score per mouse was calculated by averaging the scores of the 20 fields, resulting in an overall score between zero (no lung injury) and one (severe lung injury). The collagen score was calculated as previously described to grade lung fibrosis (37, 38). In brief, a paraffin section of lung, stained by a trichrome method, was systematically scanned in a microscope using a 10x objective. Each successive field was individually assessed for severity of interstitial fibrosis and allotted a score between 0 and 8 using a predetermined scale of severity. After examining the whole section, the mean score of all the fields was taken as the collagen score for that section and expressed correct to two decimal places.

### BAL

Upon euthanasia, the mice lungs were lavaged with 1 ml PBS four times. The recovered BAL fluids (about 3.4 ml) were centrifuged, and pelleted cells were visualized and counted on Hema 3-stained cytocentrifuge slides (39–42).

### scRNA-seq analysis

A lab protocol was developed to combine the 10x Genomics single-cell 5’ kit with customized sample multiplexing. Lungs freshly dissected from euthanized mice were dissociated into a single-cell suspension (43, 44). The cell samples of four mice from each group were pooled together and stained with TotalSeq CD45 antibody conjugated with unique oligo barcodes purchased from BioLegend. All hashtagged samples were pooled together for Gel Bead-in-Emulsion (GEM) partitioning and library construction using 10x genomics single cell 5’ kit (CG000330 Rev A). The resulting gene expression and cell hashing libraries were sequenced at a respective depth of 250 and 50 million reads with paired-ends on an Illumina NovaSeq S4 instrument. The 10x Genomics’ Cell Ranger pipeline was used to demultiplex hashtags into separate samples, perform alignment and filtering, decode cellular barcodes, count unique molecular identifiers (UMIs), normalize counts, and generate feature-barcode matrices. The derived matrices were further processed in Seurat. Quality control was firstly pursued to remove cells with low reads (UMIs<=500, Features<=200) and excess mitochondrial expression (>=12%) and batch effects, followed by dimensionality reduction, cell clustering, cell type classification, cell counting, and gene expression comparative analysis among cell clusters where differentiated gene expression was assessed at the population level and the cellular level. Differentially expressed genes (DEGs) derived above were imported into Ingenuity Pathway Analysis (IPA) (QIAGEN) for pathway and functional analysis.

scRNA-seq data in Lung Cancer Atlas (LuCA) were also analyzed to examine PDLIM2 expression in the lung macrophages of COPD patients versus normal individuals using the scANVI pipeline (45). The RDS file from the core atlas was downloaded from LuCA and imported into Seurat. Cells of interest were extracted by assay (10x 3’ v2), cell type (Macrophage), disease (Normal Control/COPD), and feature (PDLIM2). PDLIM2 expression in macrophages from each patient was calculated as the average of its cellular expression. Patients with fewer macrophage cells were skipped. Analysis of PDLIM2 expression in neutrophils was aborted as not enough cells were characterized in the original atlas data.

### Gene microarray and bulk RNA-seq analysis

Lung Genomics Research Consortium (LGRC) gene expression microarray data were analyzed to examine PDLIM2 expression in the lungs of patients with COPD or ILD. The data had been normalized using a pairwise cyclic loess approach, and the probes were collapsed to one probe per gene by selecting the probe with the highest average signal (46–49).

### *Ex vivo* phagocytosis assays

As described before (50), fresh mouse lung tissues were minced into small pieces, gently pressed with the syringe plunger top, and filtered with 40μm cell strainers. Cells passed through the strainer were seeded in a 24-well ultra-low attachment plate (Corning Inc., Corning, NY, USA) with 400μl culture medium containing the indicated antibody for 20 minutes. The cells were spun down at 1800 rpm for 5 min, and the supernatant was replaced with 400μl pHrodo Green *S. aureus* Bioparticles Conjugate (Thermo Fisher Scientific, Waltham, MA, USA. 1mg/ml) in Live Cell Imaging Solution (Thermo Fisher Scientific, Waltham, MA, USA). Two hours later, the phagocytic abilities of lung alveolar macrophages (AMs) were determined by flow cytometry (50, 51).

### *Ex vivo* NET formation assays

Bone marrow cells were flushed from the femurs of the indicated mice and pelleted via centrifugation at 427 x g for 7 minutes at 4 °C. After red blood cell lysis, neutrophils were isolated at the interface of the Histopaque 1119 and Histopaque 1077 layers via density gradient centrifugation (52). Isolated neutrophils were cultured in duplicates in a 6-well plate at a concentration of 10 million cells per well. One duplicate set was stimulated with 50 ug/mL LPS overnight, while the other served as untreated control. To quantify NET formation, 100ul of culture supernatant was aspirated post-centrifugation and transferred to a 96-well plate. The samples were stained with PicoGreen (1:10,000 dilution), and fluorescence was measured on SpectraMAX ID3. The final NET quantification was determined by subtracting the reads of the untreated controls from the reads of the LPS-treated wells.

### Statistics

Student’s *t* test (2-tailed, unpaired) was used to assess the significance of differences between 2 groups. Ordinary 1-way ANOVA was used to assess the significance of differences among groups of more than 2. All bars in the figures represent mean ± SEM. P values less than 0.05 and 0.01 were considered statistically significant and highly statistically significant, respectively.

## Results

### PDLIM2 repression in human lung diseases

**Figure 1:**
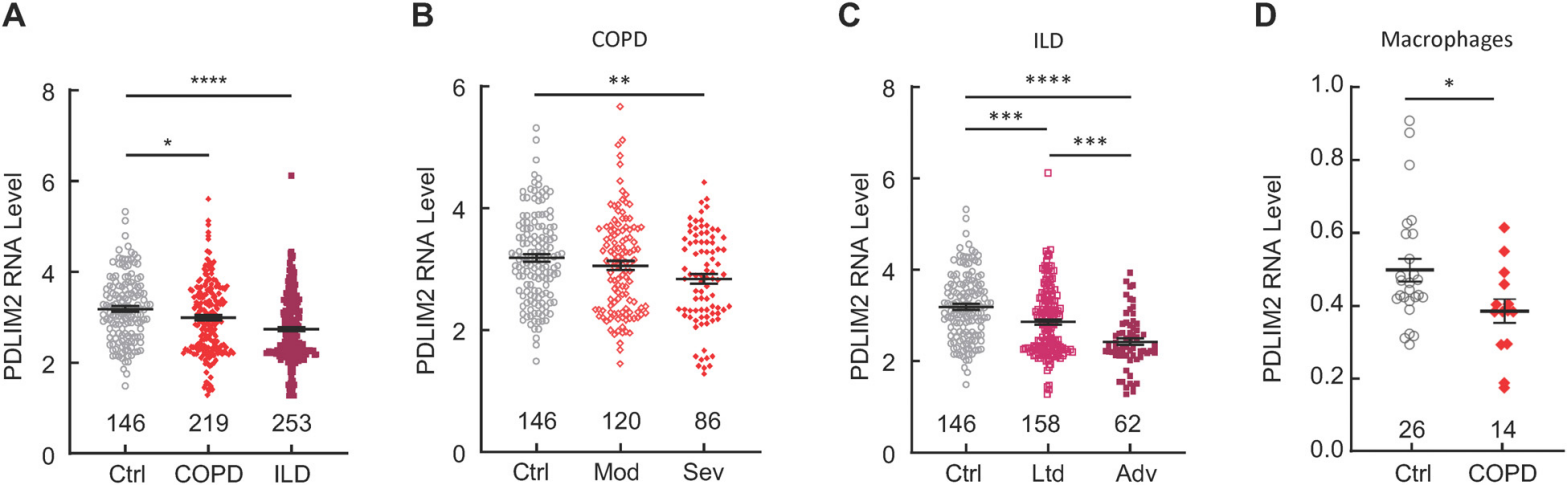
PDLIM2 repression in human COPD and ILD/IPF. **(A)** Analysis of LGRC database showing PDLIM2 repression in human COPD and ILD/IPF. **(B)** LGRC data showing association between PDLIM2 repression and COPD severity. **(C)** LGRC data showing association between PDLIM2 repression and ILD/IPF severity. **(D)** Analysis of the LuCA scRNA-seq data showing PDLIM2 repression in the lung macrophages of COPD patients. Ctrl stands for normal lung tissues. Sample numbers are listed above the x-axis. Each data point represents one patient. Data represent means ± SEM. *P < 0.05; **P < 0.01; ***P < 0.001; ****P < 0.0001; ANOVA or Student’s *t* test.

Analysis of LGRC database revealed that PDLIM2 expression was decreased in the lungs of patients with COPD or ILD/IPF (**Figure 1A**, **Supplementary Table S1**). Of note, PDLIM2 repression was associated with the severity of COPD and ILD/IPF (**Figures 1B, C**). Analysis of the scRNA-seq data in LuCA further revealed that PDLIM2 was repressed in the lung macrophages of COPD patients (**Figure 1D**). Macrophages are the most abundant immune cells within the lung and serve as the key sentinels in the lung. They are also the main culprits of various pathogenic conditions, including COPD, ILD/IPF, lung cancer, and pulmonary infectious disease (7–9, 22, 32–34, 53, 54). Currently, it remains largely unknown how the important immune cells are transformed from lung guards into perpetrators. These data identified PDLIM2 repression as a common phenotype of COPD and ILD/IPF that may cause the pathogenic transformation of lung macrophages for disease progression.

### Increased susceptibility to LPS-induced lung injury and death by myeloid PDLIM2 deletion

**Figure 2:**
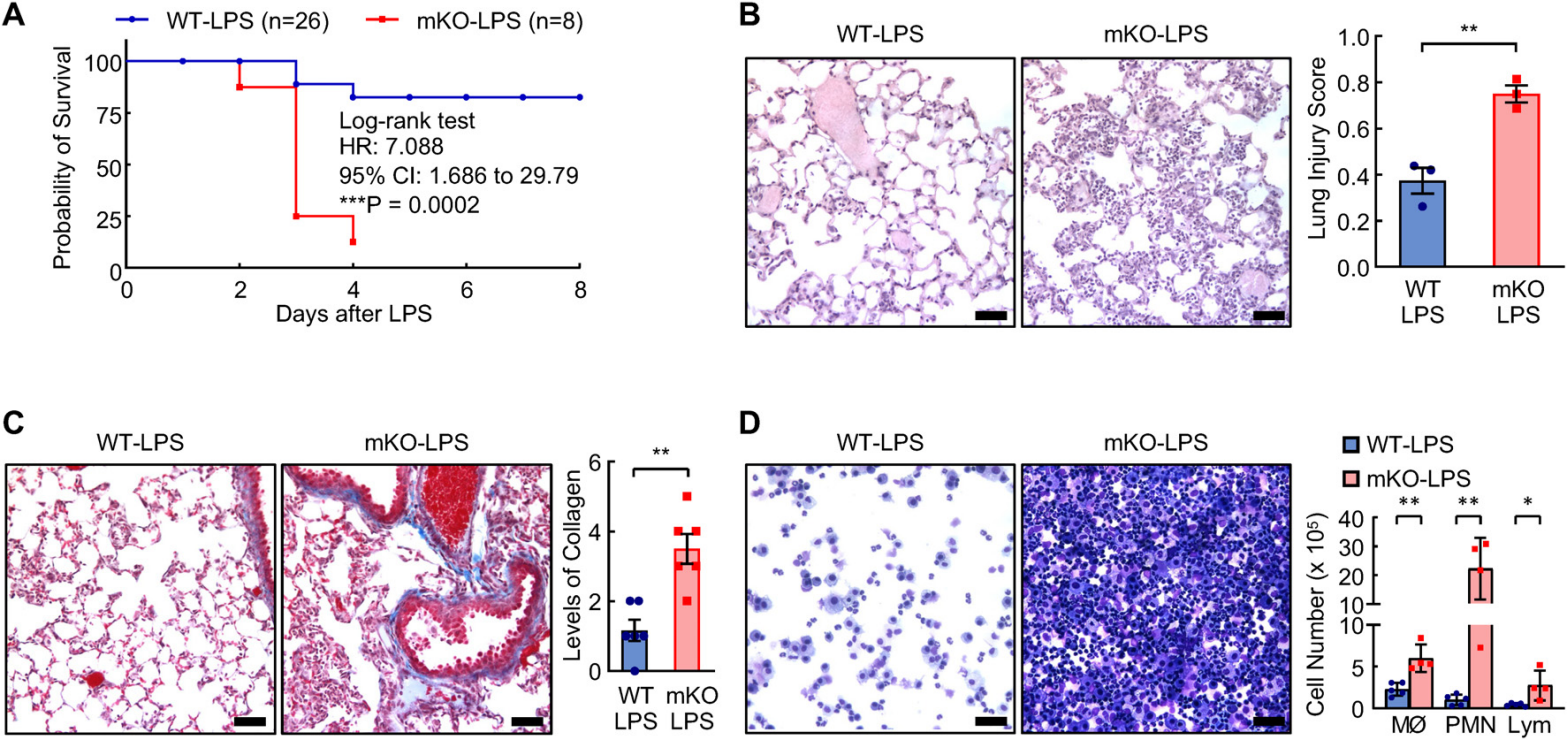
Indispensable role of myeloid PDLIM2 in preventing lung injury and death induced by LPS. **(A)** Kaplan-Meier curve showing increased mortality of PDLIM2 mKO mice treated with LPS. **(B)** H&E staining showing worsen lung damage and augmented immune cell lung infiltration in PDLIM2 mKO mice treated with LPS (n = 3). **(C)** Trichrome staining showing higher collagen accumulation in the lung of PDLIM2 mKO mice treated with LPS (n = 6). **(D)** Hema 3 staining of BAL cells on cytocentrifuge slides showing more immune cells in the lung of PDLIM2 mKO mice treated with LPS (WT: n = 5; mKO: n = 4). Scale bars: 25 µm. Data represent means ± SEM. *P < 0.05; **P < 0.01; Student’s *t* test.

To examine the role of lung macrophage intrinsic PDLIM2 in lung diseases, PDLIM2 mKO mice, in which PDLIM2 is selectively deleted in lung macrophages and other myeloid cells, were subjected to LPS model of ALI/ARDS (55). This model can also provide mechanistic insights into COPD, PF and lung infectious disease (54–57). In consistent with previous studies (58), LysM-Cre^+/-^ mice showed no difference in LPS-induced ALI as FVB/N mice and PDLIM2^flox/flox^ mice, all of which have normal PDLIM2 expression and are included in the WT control group of the studies (**Figure 2A**). PDLIM2 mKO mice showed markedly decreased survival after LPS treatment at the dose of 4 mg/kg body weight, compared to the WT control mice. Consistently, the lung tissues of PDLIM2 mKO mice showed exacerbated lung injury and much more severe inflammatory reactions, including inflammatory cell infiltration, alveolar congestion, alveolar wall thickening, hemorrhage, and collagen accumulation (**Figures 2B–D**). These data suggested that PDLIM2 repression, particularly in lung macrophages and neutrophils, is a key mechanism driving pathogenic inflammation and rendering patients much more vulnerable to infection, lung damage, and mortality.

### Imbalanced activation of pro- versus anti- inflammatory signaling pathways in the total lung immune cell population of myeloid PDLIM2 deletion mice by LPS

To systematically define the molecular mechanisms underlying the super-sensitivity of PDLIM2 mKO mice to LPS, immune cells enriched from the lung tissues of PDLIM2 mKO and WT mice treated with LPS or PBS were subjected to scRNA-seq. In line with no abnormalities of PDLIM2 mKO mice under pathogen-free conditions, scRNA-seq data showed no significant difference in the numbers and composition of immune cells, including macrophages and neutrophils in the lung of PDLIM2 mKO and WT mice treated with PBS (**Supplementary Figure S1**). As expected, LPS treatment induced dramatic but overall similar changes in both PDLIM2 mKO and WT mice. The top upstream regulators activated by LPS are also the same in both mice (**Supplementary Figure S2**).

**Figure 3:**
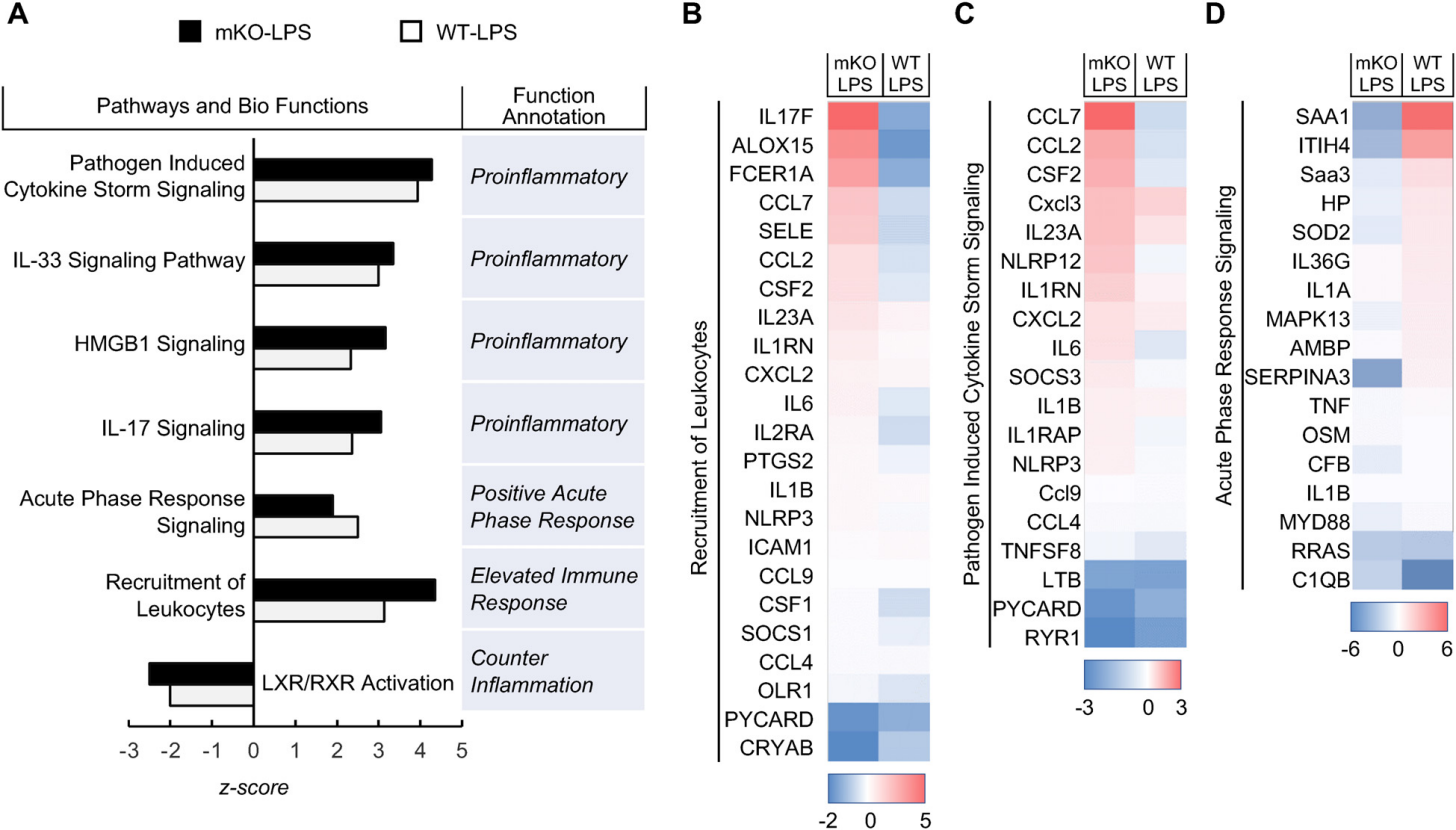
Over-activated pro-inflammatory signaling pathways in total lung immune cell population of LPS-treated myeloid PDLIM2 deletion mice. **(A)** Pathway and function analysis showing higher activated pro-inflammatory signaling pathways but less activated or more repressed anti-inflammatory signaling pathways in whole immune cells in the lung of LPS-treated PDLIM2 mKO mice. **(B)** Gene expression heatmap of component genes in lung immune cells showing increased leukocyte recruitment activation in the lung of LPS-treated PDLIM2 mKO mice. **(C)** Gene expression heatmap of component genes in lung immune cells showing increased pathogen-induced cytokine storm signaling in the lung of LPS-treated PDLIM2 mKO mice. **(D)** Gene expression heatmap of component genes in lung immune cells showing decreased acute phase response signaling in the lung of LPS-treated PDLIM2 mKO mice.

Consistent with the histological studies above, notably, the scRNA-seq data indicated that LPS treatment led to more macrophages, neutrophils, T and B cells in the lung of PDLIM2 mKO mice in comparison to WT mice (**Supplementary Figure S1D**). In further support of this, the pathways and functions for leukocyte recruitment were activated by LPS at a higher level in all pulmonary immune cells of PDLIM2 mKO mice as a whole population (**Figures 3A, B**). Furthermore, the inflammatory signaling pathways were more activated at the total population level of lung immune cells in the PDLIM2 mKO mice, as evidenced by the higher activated pathogen-induced cytokine storm signaling, IL-17 signaling, IL-33 signaling, and HMGB1 signaling (**Figures 3A, C**). In contrast, activation of the acute phase response (APR) signaling pathway, which plays a central role in restoring tissue homeostasis, was activated at lower levels (**Figures 3A, D**). On the other hand, the anti-inflammatory LXR/RXR signaling pathway was repressed to a greater extent (**Figure 3A**). Similar patterns were seen for these signaling pathways in the lung of patients with COPD, ILD, or ALI/ARDS (**Supplementary Figure S3**). These data suggested that PDLIM2 repression, particularly in lung myeloid cells, disrupts the immune balance toward excessive inflammation in the lung in response to lung infection.

### Overactive NF-κB and pathogenic activation of lung macrophages in myeloid PDLIM2 deletion mice by LPS

**Figure 4:**
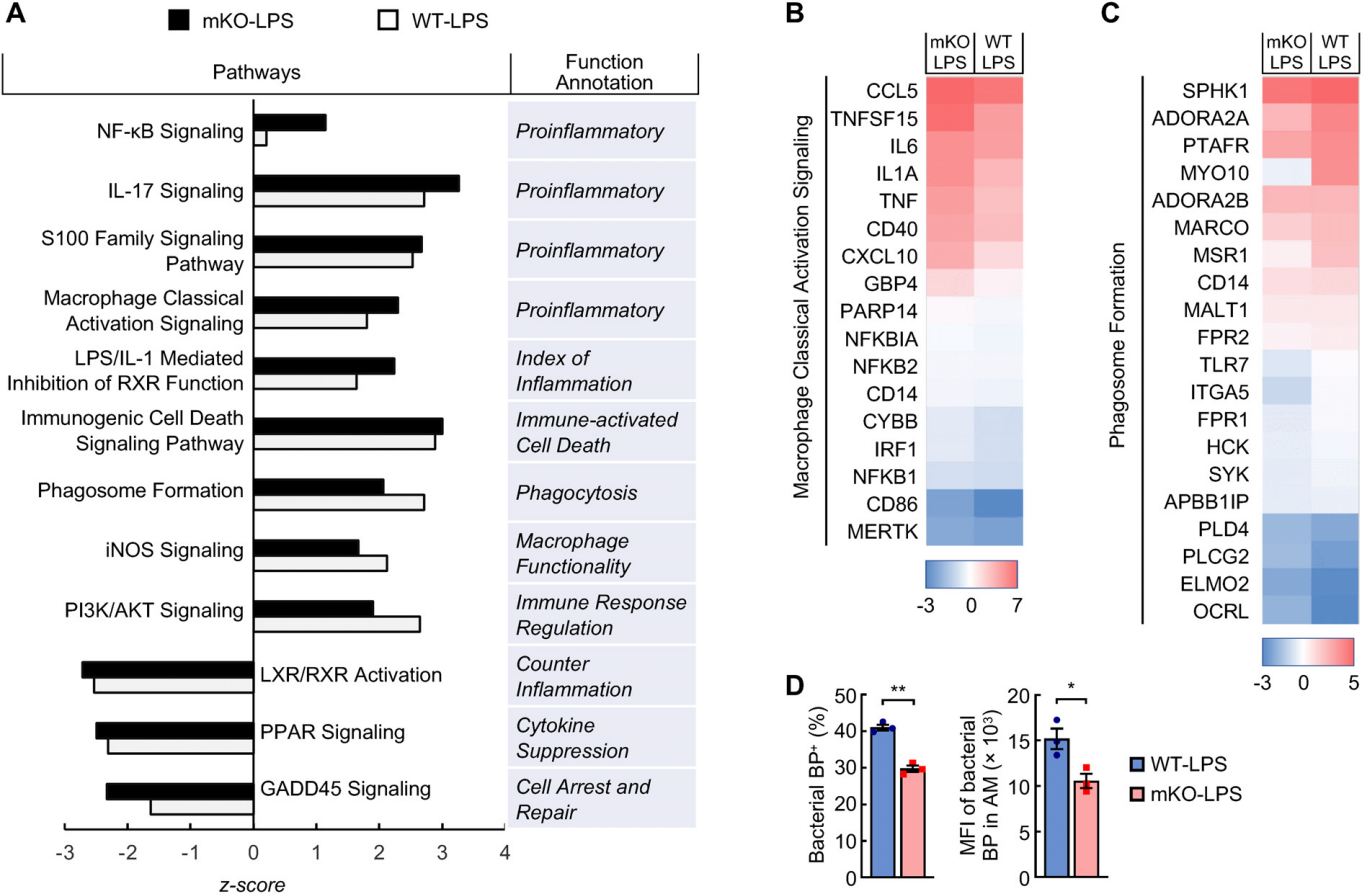
Pathogenic activation of signaling pathways in the lung macrophages of LPS-treated myeloid PDLIM2 deletion mice. **(A)** Pathway and function analysis of lung macrophages showing hyper-activated pro-inflammatory signaling pathways but less activated or more repressed signaling pathways involved in anti-inflammation or macrophage innate immunity function in LPS-treated PDLIM2 mKO mice. **(B)** Gene expression heatmap of component genes showing increased macrophage classical activation signaling pathway in the lung macrophages of LPS-treated PDLIM2 mKO mice. **(C)** Gene expression heatmap of component genes showing lower phagosome formation ability of the lung macrophages in LPS-treated PDLIM2 mKO mice. **(D)***Ex vivo* phagocytosis assays showing defective ability of AMs from LPS-treated PDLIM2^mKO^ mice in phagocytizing pHrodo green *S. aureus* bioparticles (BP) (n = 3). Data represent means ± SEM. *P < 0.05; **P < 0.01; Student’s *t* test.

Logically, the phenotypes in LPS-treated PDLIM2 mKO mice were originally initiated and mostly attributed to PDLIM2 deficiency in lung macrophages, because they are the first immune cells that encounter LPS. Thus, the molecular difference of lung macrophages in LPS-treated PDLIM2 mKO and WT mice was characterized. In line with the role of PDLIM2 in repressing the master pro-inflammation transcription factor NF-κB (19–25, 59–66), scRNA-seq data showed much higher activation of the NF-κB signaling pathway in the lung macrophages of PDLIM2 mKO mice treated with LPS (**Figure 4A**). The proinflammatory macrophage classical activation signaling pathway was also more activated in the lung macrophages of LPS-treated PDLIM2 mKO mice (**Figures 4A, B**). However, the phagosome formation and inducible nitric oxide synthetase (iNOS) signaling pathways, which are critical for the most important innate immune function of macrophages to directly kill and clear pathogens, were less activated (**Figures 4A, C**). Consistently, AMs from LPS-treated PDLIM2 mKO mice showed a reduced phagocytotic ability *ex vivo* (**Figure 4D**).

Many other inflammation pathways were also found to be more activated in the lung macrophages of the LPS-treated PDLIM2 mKO mice, such as IL-17 signaling, LPS/IL-1 mediated inhibition of RXR function, S100 family signaling, and immunogenic cell death signaling (**Figure 4A**). In sharp contrast, several signaling pathways known to counter and repress inflammation and/or facilitate the innate immunity and wound repairing of macrophages, i.e., PI3K/AKT, LXR/RXR, PPAR, and GADD45 signaling, were either less activated or more repressed. These data suggested that PDLIM2 repression leads to NF-κB over-activation induced by LPS, eventually resulting in aberrant activation of lung macrophages for pathogenic inflammation and fatal lung injury.

### Exaggerated NF-κB activity and divergent activation of lung neutrophils in myeloid PDLIM2 deletion mice by LPS

**Figure 5:**
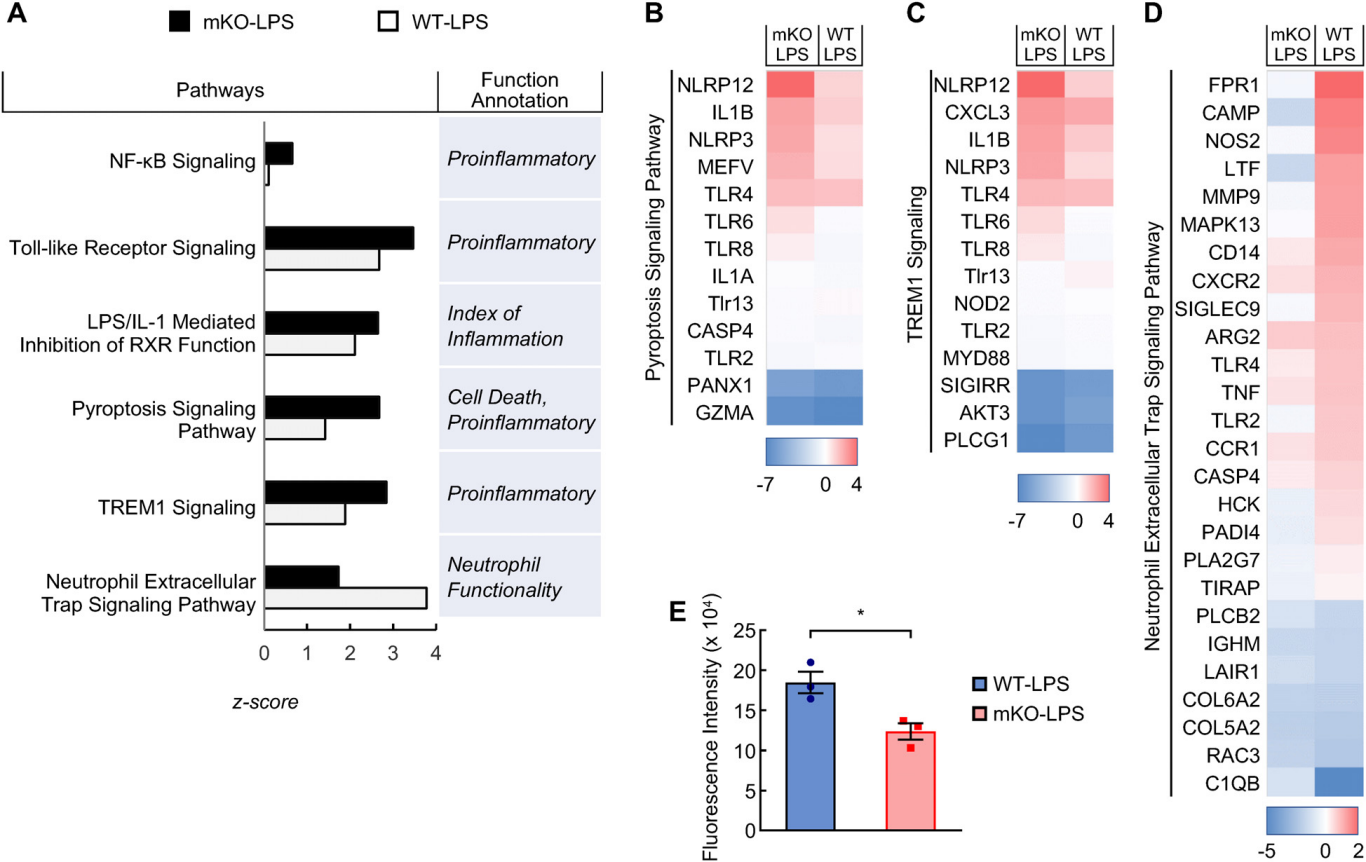
Aberrant activation of signaling pathways in the lung neutrophils of LPS-treated myeloid PDLIM2 deletion mice. **(A)** Pathway and function analysis of lung neutrophils showing superior activated pro-inflammatory signaling pathways but less activated signaling pathways involved in neutrophil innate immunity function in LPS-treated PDLIM2 mKO mice. **(B)** Gene expression heatmap of component genes showing elevated activation of the pyroptosis signaling pathway in the lung neutrophils of LPS-treated PDLIM2 mKO mice. **(C)** Gene expression heatmap of component genes showing elevated TREM1 signaling activation in the lung neutrophils of LPS-treated PDLIM2 mKO mice. **(D)** Gene expression heatmap of component genes showing less activated NET signaling in the lung neutrophils in LPS-treated PDLIM2 mKO mice. **(E)** Ex vivo NET assays showing defective NET ability of LPS-treated neutrophils deficient in PDLIM2 (n = 3). Data represent means ± SEM. *P < 0.05; Student’s t test.

Following lung macrophage activation by pulmonary infection, neutrophils are the first to be recruited into the lung and become predominant. Like macrophages, neutrophils are major lung sentinels that could also be pathogenic drivers. Consistent with their PDLIM2 deficiency, neutrophils in the lung of LPS-treated PDLIM2 mKO mice, like lung macrophages, had much higher activation of the NF-κB signaling pathway in comparison to WT mice (**Figure 5A**). Several common pro-inflammatory signaling pathways, including toll-like receptor, LPS/IL-1 mediated inhibition of RXR function, and pyroptosis signaling pathways, were more activated in the neutrophils of the PDLIM2 mKO mice (**Figures 5A, B**). Activation of the triggering receptor expressed on myeloid cells 1 (TREM1) signaling pathway was also much higher (**Figures 5A, C**). TREM1 is predominantly expressed on neutrophils, and its activation leads to proinflammatory immune responses (67). However, the neutrophil extracellular trap (NET) signaling pathway was dramatically lower (**Figures 5A, D**). NET is an important mechanism that neutrophils use to immobilize and kill pathogens (68). In support of this, PDLIM2-deficient neutrophils treated with LPS exhibited a reduced NET formation ability *ex vivo* in comparison with WT neutrophils with the same LPS treatment (**Figure 5E**). These data suggested that PDLIM2 repression in neutrophils dampens their host-protective functions against lung infection and unleashes their pro-inflammatory activity to cause excessive inflammation and lethal lung injury.

## Discussion

The studies above provide the first evidence linking PDLIM2 to ALI/ARDS, COPD, ILD/IPF, and lung infection and infectious disease. As a matter of fact, they are the first investigation on the role of PDLIM2 in the lung diseases other than lung cancer. They have identified PDLIM2 repression as a common phenomenon of human COPD, ILD/IPF, and lung cancer. Using the LPS mouse model, they have further shown that repression of PDLIM2 in myeloid cells, especially in lung macrophages, is a causal mechanism underlying infection-induced ALI/ARDS and death.

PDLIM2 protects against lung infection by limiting inflammation from damage while simultaneously promoting pathogen killing (**Supplementary Figure S4**). PDLIM2 repression in lung macrophages and neutrophils results in uncontrolled activation of NF-κB in these crucial immune cells, thereby unleashing their pro-inflammatory activity and dampening their host-protective functions against lung infection. The pathogenic activation of lung macrophages and neutrophils causes excessive inflammation in the lung and subsequently lung damage and animal death.

It seems that myeloid PDLIM2 repression is dispensable for or only plays an insufficient role in the initiation or even the early stages of COPD and ILD/IPF, since PDLIM2 mKO mice are healthy and show no phenotypes under pathogen-free conditions, including no COPD and ILD/IPF development. Nevertheless, PDLIM2 expression is largely intact in the lung of COPD and ILD/IPF patients at the early disease stages. Except for rendering patients vulnerable to lung infections, however, the aberrant inflammation caused by PDLIM2 repression may also contribute to COPD, ILD/IPF lung disease progression, as suggested by the association of PDLIM2 repression with the severity of COPD, ILD/IPF in humans and the excessive lung inflammation and damage caused by myeloid PDLIM2 deficiency in mice treated with LPS, which in COPD, ILD/IPF may also cause disease progression.

Recent studies indicate that PDLIM2 repression in the lung macrophages of mice with lung cancer is attributed to the transcription repressor BACH1 activated by reactive oxygen species (ROS) (22). Given the high ROS levels in the lung of patients with COPD and ILD/IPF (69), it is highly plausible that PDLIM2 repression in COPD and ILD/IPF is also mediated by ROS-activated BACH1. Interestingly, ROS inhibitors can restore PDLIM2 expression in lung macrophages and prevents lung cancer *in vivo* (22). Furthermore, delivery of exogenous PDLIM2 by clinically feasible nanoparticles shows high therapeutic efficacy in the mouse model of refractory lung cancer (21). Thus, therapeutic strategies for targeting PDLIM2 should be further explored for the prevention and treatment of COPD, ILD/ILD, ALI/ARDS, and infectious disease.

## Data Availability

From https://www.ncbi.nlm.nih.gov/projects/gap/cgi-bin/study.cgi?study_id=phs000624.v1.p1, we obtained LGRC data on human COPD and ILD/IPF. The scRNA-seq data on human COPD were obtained from LuCA at https://cellxgene.cziscience.com/collections/edb893ee-4066-4128-9aec2785eb2b03f8287. All data generated or analyzed during this study are included in this article and its additional files. The mouse scRNA-seq data produced in this paper are deposited in the online NCBI Gene Expression Omnibus (GSE249800) for free access. The Extra data that support the findings of this study are available from the corresponding authors upon reasonable request.

## Supplementary material

Supplementary Figure 1 Experimental design and outline on multiplexed scRNA-seq data analysis. **(A)** Overall experimental design. **(B)** Demultiplexing of hashtagged samples. **(C)** Cell population profiling of immune cells in the lung of PDLIM2 mKO and WT mice treated with LPS or PBS. **(D)** Cell population profiling showing increased immune cells in the lung of LPS-treated PDLIM2 mKO mice.

Supplementary Figure 2 Top upstream regulators identified for LPS-induced gene expression changes in lung immune cells of PDLIM2 mKO and WT mice.

Supplementary Figure 3 Top pathways altered in the lung of patients with ILD, COPD or ARDS.

Supplementary Figure 4 Graphic summary of PDLIM2’s dual roles in the case of lung infection.

Supplementary Table 1 The clinical attributes data, downloaded from the Lung Genomics Research Consortium (LGRC) website, for all patients included in the analyses shown in **Figures 1A–C**.
