# Supplementary figures and images for: Myeloid PDLIM2 repression as a common mechanism of infection susceptibility in lung diseases
Source: Front Immunol. 2025 Nov 19;16:1669117. doi: 10.3389/fimmu.2025.1669117 (PMC12672342; doi:10.3389/fimmu.2025.1669117)

**Figure S1**

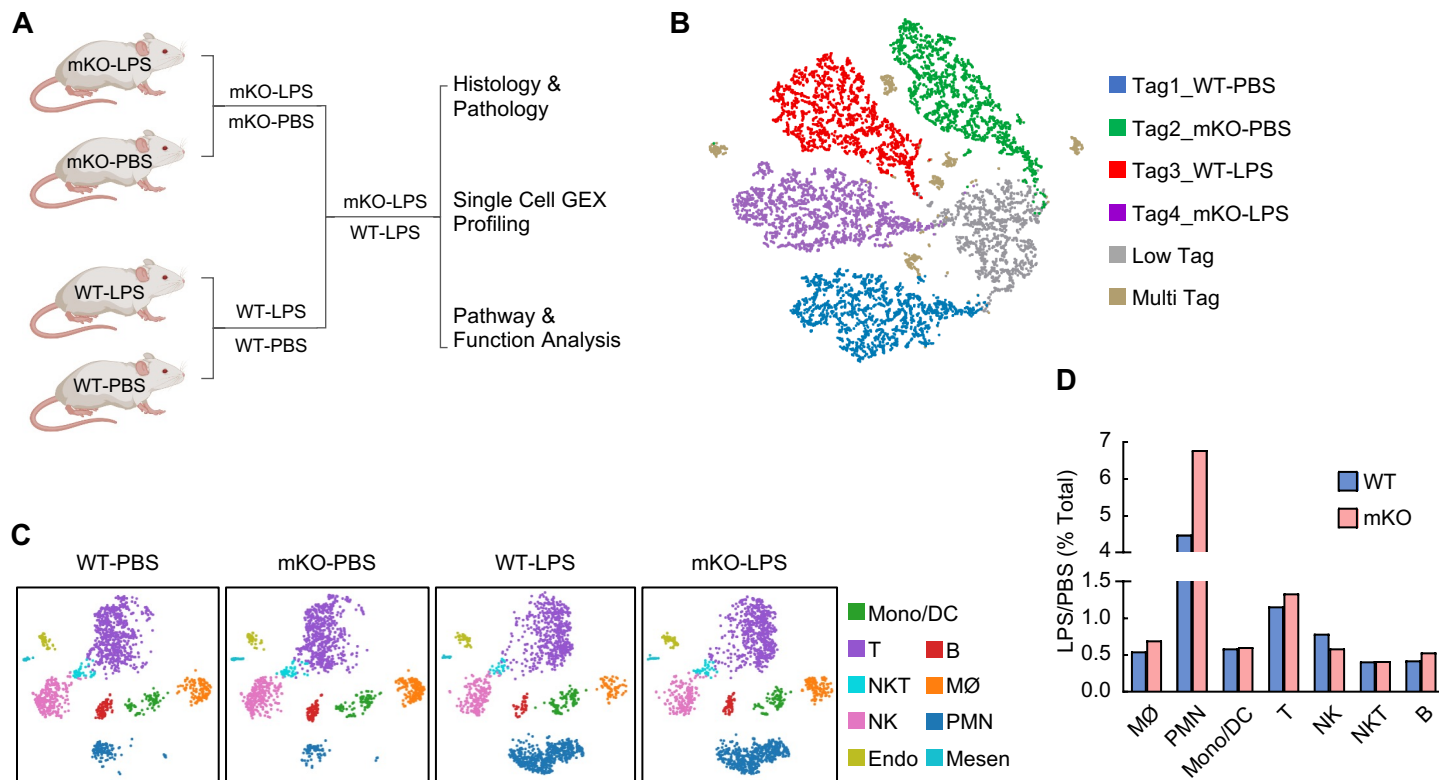

**Figure S2**

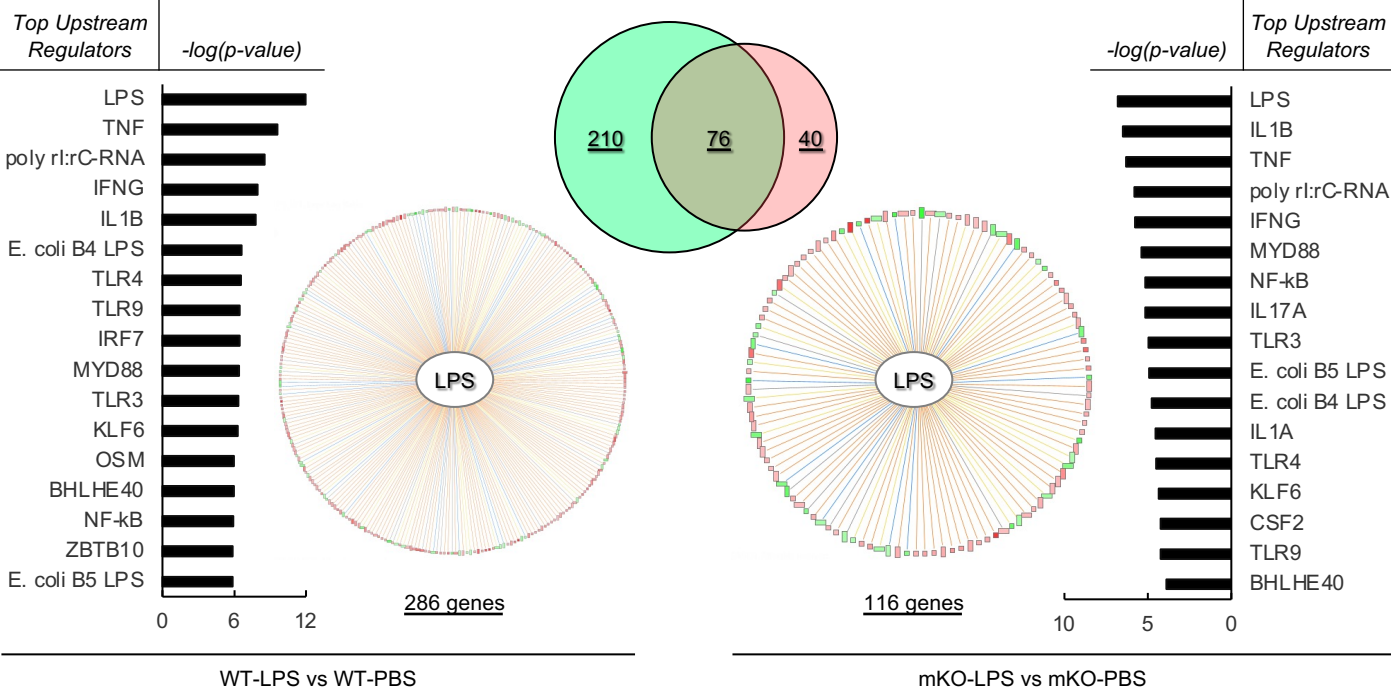

Figure S3

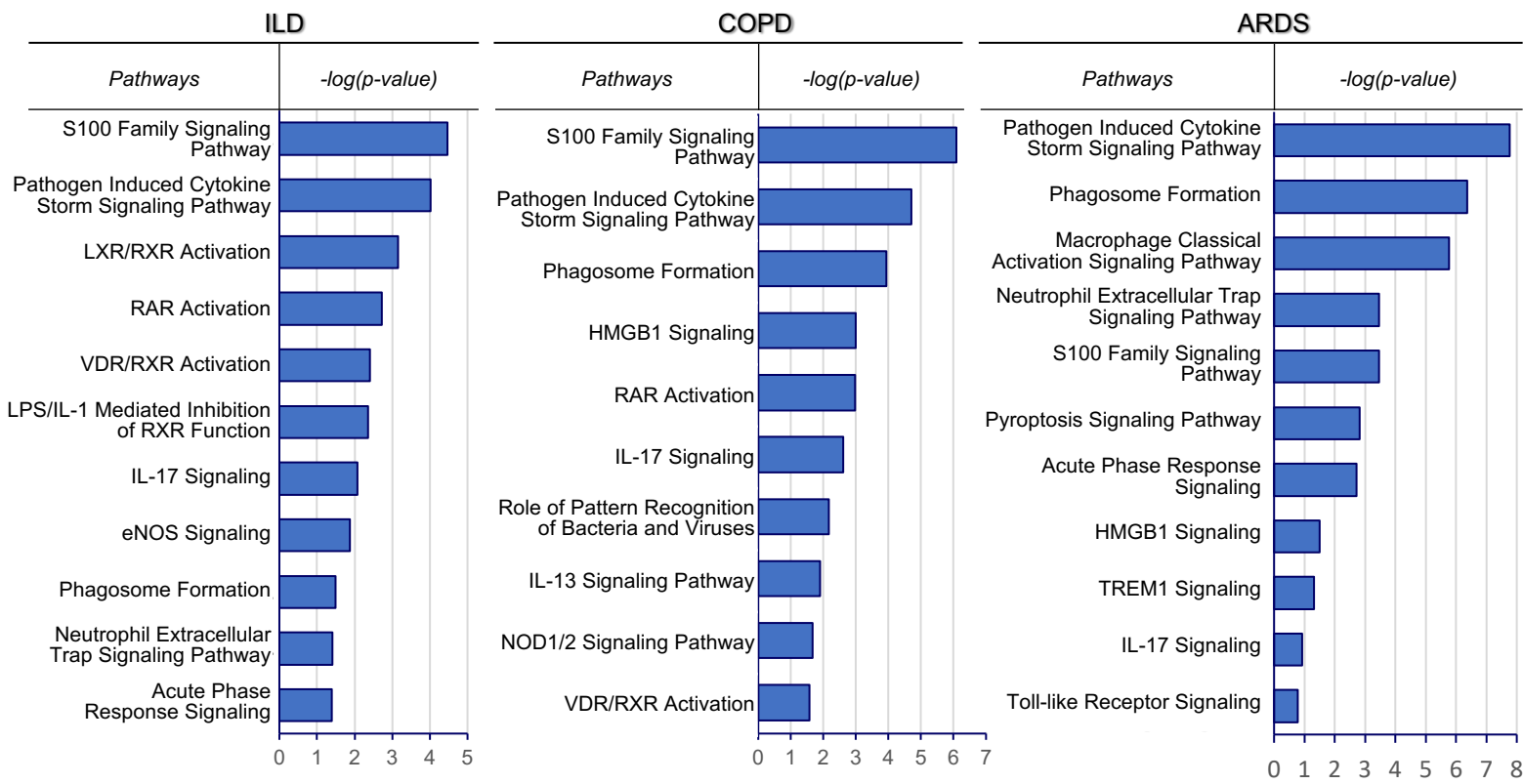

Figure S4

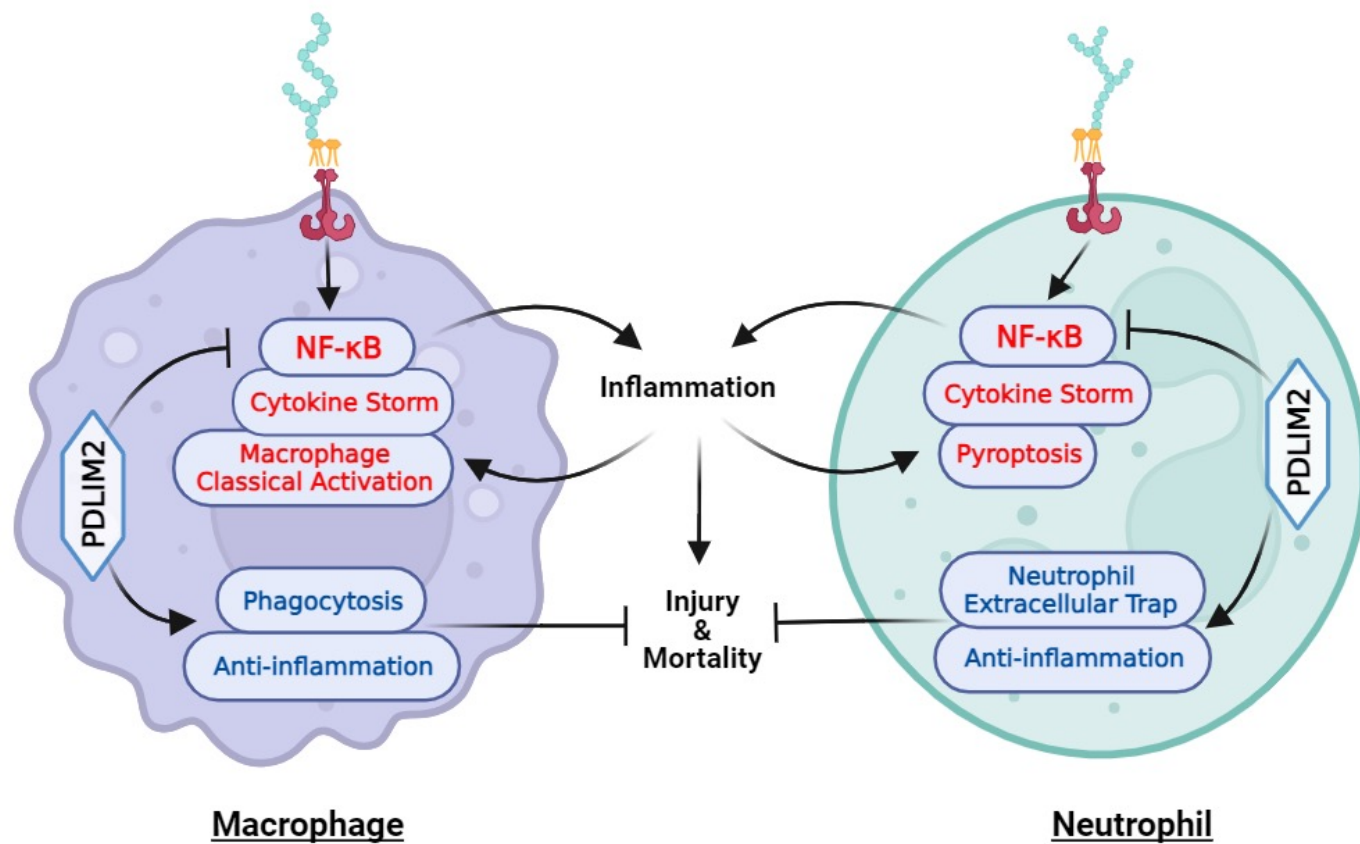

Supplement: Supplementary Figure 1 — Experimental design and outline on multiplexed scRNA-seq data analysis. (A) Overall experimental design. (B) Demultiplexing of hashtagged samples. (C) Cell population profiling of immune cells in the lung of PDLIM2 mKO and WT mice treated with LPS or PBS. (D) Cell population profiling showing increased immune cells in the lung of LPS-treated PDLIM2 mKO mice. [file DataSheet1.pdf]
